# Supplementary material for: Robot-Assisted Minimally Invasive Esophagectomy versus Open Esophagectomy for Esophageal Cancer: A Systematic Review and Meta-Analysis
Source: Cancers (Basel). 2022 Jun 29;14(13):3177. doi: 10.3390/cancers14133177 (PMC9264782; doi:10.3390/cancers14133177)

Supplemental Table S1. PRISMA 2009 Checklist.

Section/topic	#	Checklist item	Reported on page #
**TITLE**			
Title	1	Identify the report as a systematic review, meta-analysis, or both.	Manuscript Page 1
**ABSTRACT**			
Structured summary	2	Provide a structured summary including, as applicable: background; objectives; data sources; study eligibility criteria, participants, and interventions; study appraisal and synthesis methods; results; limitations; conclusions and implications of key findings; systematic review registration number.	Manuscript Pages 1
**INTRODUCTION**			
Rationale	3	Describe the rationale for the review in the context of what is already known.	Manuscript Page 2
Objectives	4	Provide an explicit statement of questions being addressed with reference to participants, interventions, comparisons, outcomes, and study design (PICOS).	Manuscript Page 2
**METHODS**			
Protocol and registration	5	Indicate if a review protocol exists, if and where it can be accessed (e.g., Web address), and, if available, provide registration information including registration number.	Not applicable
Eligibility criteria	6	Specify study characteristics (e.g., PICOS, length of follow-up) and report characteristics (e.g., years considered, language, publication status) used as criteria for eligibility, giving rationale.	Manuscript Pages 2-3
Information sources	7	Describe all information sources (e.g., databases with dates of coverage, contact with study authors to identify additional studies) in the search and date last searched.	Manuscript Page 3, Figure 1
Search	8	Present full electronic search strategy for at least one database, including any limits used, such that it could be repeated.	Manuscript Page 3, Figure 1
Study selection	9	State the process for selecting studies (i.e., screening, eligibility, included in systematic review, and, if applicable, included in the meta-analysis).	Manuscript Page 3, Figure 1
Data collection process	10	Describe method of data extraction from reports (e.g., piloted forms, independently, in duplicate) and any processes for obtaining and confirming data from investigators.	Manuscript Page 3
Data items	11	List and define all variables for which data were sought (e.g., PICOS, funding sources) and any assumptions and simplifications made.	Manuscript Pages 3-4, Tables 1,2
Risk of bias in individual studies	12	Describe methods used for assessing risk of bias of individual studies (including specification of whether this was done at the study or outcome level), and how this information is to be used in any data synthesis.	Manuscript Page 4
Summary measures	13	State the principal summary measures (e.g., risk ratio, difference in means).	Manuscript Page 4
Synthesis of results	14	Describe the methods of handling data and combining results of studies, if done, including measures of consistency (e.g., I^2) for each meta-analysis.	Manuscript Page 4
Risk of bias across studies	15	Specify any assessment of risk of bias that may affect the cumulative evidence (e.g., publication bias, selective reporting within studies).	Manuscript Page 4
Additional analyses	16	Describe methods of additional analyses (e.g., sensitivity or subgroup analyses, meta-regression), if done, indicating which were pre-specified.	Not applicable
**RESULTS**			
Study selection	17	Give numbers of studies screened, assessed for eligibility, and included in the review, with reasons for exclusions at each stage, ideally with a flow diagram.	Manuscript Page 5, Figure 1
Study characteristics	18	For each study, present characteristics for which data were extracted (e.g., study size, PICOS, follow-up period) and provide the citations.	Manuscript Page 5, Table 1

Risk of bias within studies	19	Present data on risk of bias of each study and, if available, any outcome level assessment (see item 12).	Manuscript Pages 5-8, Supplemental Tables 3-4
Results of individual studies	20	For all outcomes considered (benefits or harms), present, for each study: (a) simple summary data for each intervention group (b) effect estimates and confidence intervals, ideally with a forest plot.	Manuscript Pages 5-8, Figures 2-6, Table 2
Synthesis of results	21	Present results of each meta-analysis done, including confidence intervals and measures of consistency.	Manuscript Pages 5-8, Figures 2-6, Table 2
Risk of bias across studies	22	Present results of any assessment of risk of bias across studies (see Item 15).	Manuscript Page 5, Supplemental Figures 1-2
Additional analysis	23	Give results of additional analyses, if done (e.g., sensitivity or subgroup analyses, meta-regression [see Item 16]).	Not applicable
**DISCUSSION**			
Summary of evidence	24	Summarize the main findings including the strength of evidence for each main outcome; consider their relevance to key groups (e.g., healthcare providers, users, and policy makers).	Manuscript Pages 20-22
Limitations	25	Discuss limitations at study and outcome level (e.g., risk of bias), and at review-level (e.g., incomplete retrieval of identified research, reporting bias).	Manuscript Pages 22
Conclusions	26	Provide a general interpretation of the results in the context of other evidence, and implications for future research.	Manuscript Pages 22
**FUNDING**			
Funding	27	Describe sources of funding for the systematic review and other support (e.g., supply of data); role of funders for the systematic review.	Not applicable

Supplemental Table S2. Quality assessment of included non-randomized studies utilizing the Newcastle–Ottawa scale.

Study (Author, Year)	Representativeness of RAMIE	Selection of OE	Ascertainment of Exposure	Demonstration That Outcome of Interest Was Not Present at Start of Study	Comparability on Overall Morbidity	Comparability on 90-days Mortality	Assessment of Outcome	Long Enough Follow-up (≥90 days)	Adequacy (≥90%) of Follow-up	Total score
**Gong et al., 2020**	1	1	1	1	1	1	1	1	1	9
**Sugawara et al., 2020**	1	1	1	1	0	0	1	1	1	7
**Sarkaria et al., 2019**	1	1	1	1	0	1	1	1	1	8
**Yun et al., 2019**	1	1	1	1	0	0	1	1	1	7
**Meredith et al., 2019**	1	1	1	1	1	1	1	1	1	9
**Osaka et al., 2018**	1	1	1	1	0	0	1	0	0	5
**Jeong et al., 2016**	1	1	1	1	1	0	1	0	0	6
**Mori et al., 2016**	1	1	1	1	0	0	1	0	0	5
**Diez del Val et al., 2015**	1	1	1	1	1	0	1	0	0	6

RAMIE: Robotic-assisted minimally invasive esophagectomy; OE: Open esophagectomy.

Supplemental Table S3. Quality assessment of included randomized controlled trials utilizing the Cochrane Collaboration's tool.

Study (Author, year)	Selection bias		Performance bias	Detection bias	Attrition bias	Reporting bias	
	Random Sequence Generation	Allocation concealment	Blinding of participants and personnel	Blinding of outcome assessment	Incomplete outcome data	Selective reporting	Other bias
**Van der Sluis et al., 2019**	LR	LR	N/A	N/A	LR	LR	LR

LR: Low Risk; N/A: non-applicable.

Supplemental Figure S1. Funnel plots to assess publication bias (primary surgical and primary oncological outcomes), Supplemental

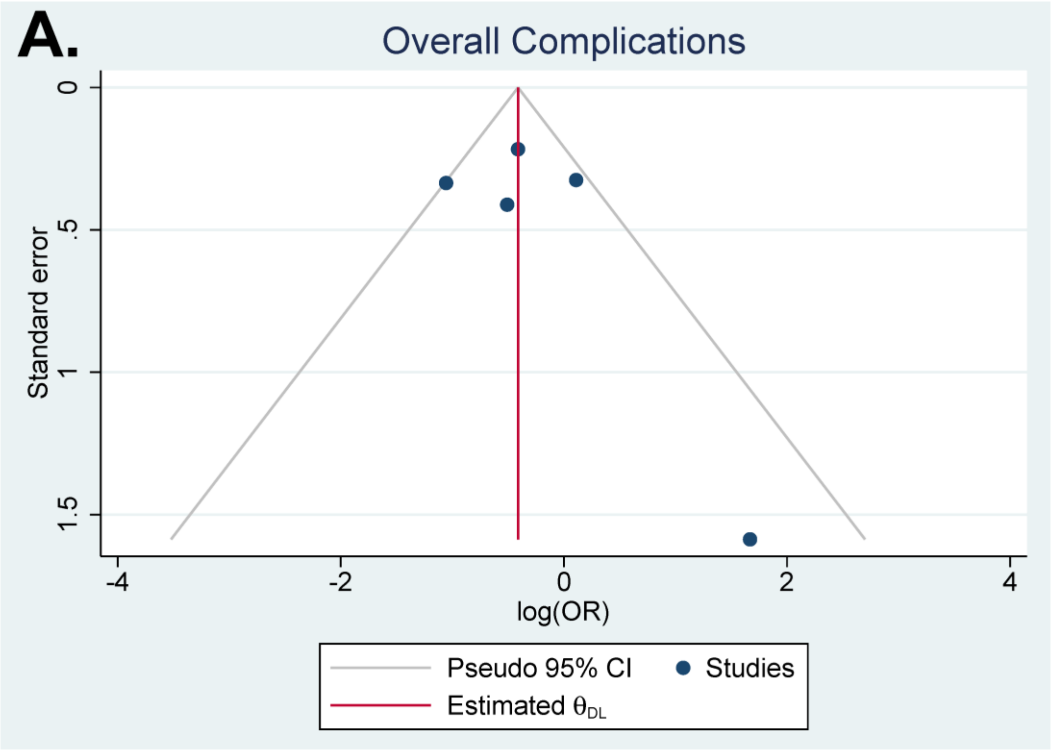

Supplemental Figure S2. Funnel plots to assess publication bias (secondary outcomes).

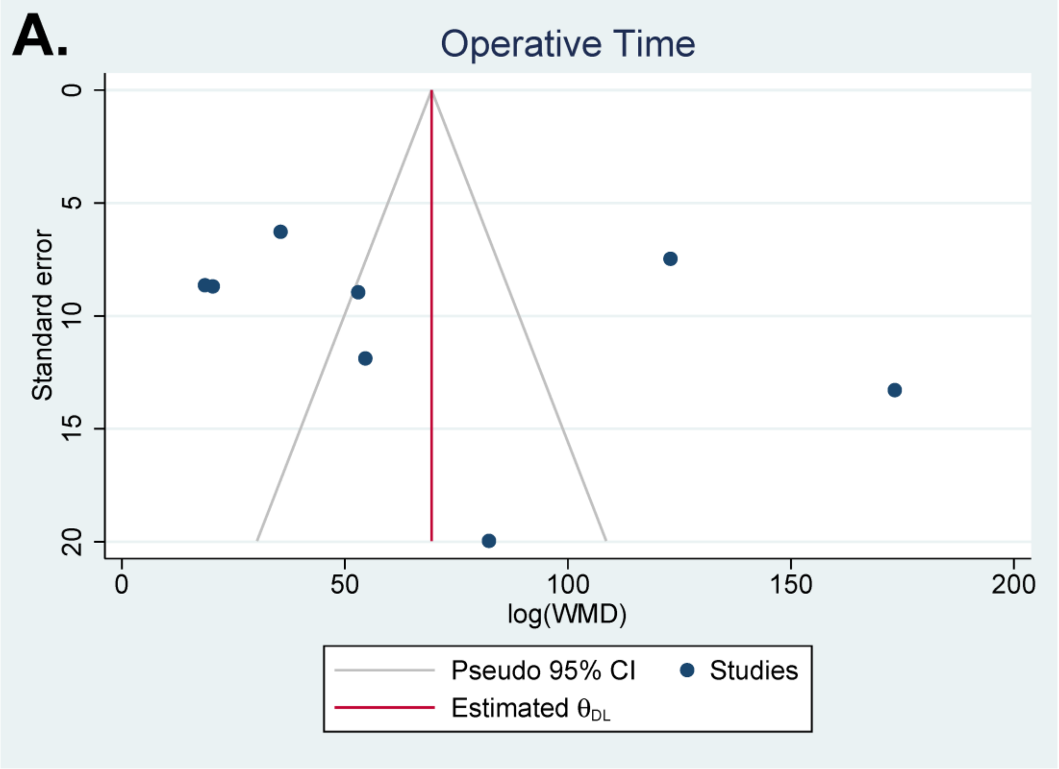

Supplement: Supplementary file 1 [file cancers-14-03177-s001.zip › cancers-1621673 supplementary.pdf]
